# Biochemical composition of temperate and Arctic populations of *Saccharina latissima* after exposure to increased pCO_2_ and temperature reveals ecotypic variation

**DOI:** 10.1007/s00425-014-2143-x

**Published:** 2014-08-26

**Authors:** Mark Olischläger, Concepción Iñiguez, Francisco Javier López Gordillo, Christian Wiencke

**Affiliations:** 1 Department of Functional Ecology, Alfred-Wegener-Institute, Helmholtz Center for Marine and Polar Research, Am Handelshafen 12, 27570 Bremerhaven, Germany; 2 Department of Ecology, Faculty of Sciences, University of Malaga, Bulevar Louis Pasteur s/n, 29010 Málaga, Spain

**Keywords:** Chemical composition, CO_2_, DIC, Ecotype, Global change, Macroalgae, *Saccharina*, Temperature

## Abstract

Previous research suggested that the polar and temperate populations of the kelp *Saccharina latissima* represent different ecotypes. The ecotypic differentiation might also be reflected in their biochemical composition (BC) under changing temperatures and pCO_2_. Accordingly, it was tested if the BC of Arctic (Spitsbergen) and temperate *S. latissima* (Helgoland) is different and if they are differently affected by changes in temperature and pCO_2_. Thalli from Helgoland grown at 17 °C and 10 °C and from Spitsbergen at 10 °C and 4 °C were all tested at either 380, 800, or 1,500 µatm pCO_2_, and total C-, total N-, protein, soluble carbohydrate, and lipid content, as well as C/N-ratio were measured. At 10 °C, the Arctic population had a higher content of total C, soluble carbohydrates, and lipids, whereas the N- and protein content was lower. At the lower tested temperature, the Arctic ecotype had particularly higher contents of lipids, while content of soluble carbohydrates increased in the Helgoland population only. In Helgoland-thalli, elevated pCO_2_ caused a higher content of soluble carbohydrates at 17 °C but lowered the content of N and lipids and increased the C/N-ratio at 10 °C. Elevated pCO_2_ alone did not affect the BC of the Spitsbergen population. Conclusively, the Arctic ecotype was more resilient to increased pCO_2_ than the temperate one, and both ecotypes differed in their response pattern to temperature. This differential pattern is discussed in the context of the adaptation of the Arctic ecotype to low temperature and the polar night.

## Introduction

Brown algae of the order Laminariales (kelps) often dominate the sublittoral zone of rocky shores in temperate and polar environments (Lüning 1990) and provide food and habitat for a great number of associated organisms (Bartsch et al. 2008). Economically, the Laminariales are of interest since they are cultivated in large quantities for human nutrition (Bartsch et al. 2008). The Laminariales in general, but the species *Saccharina latissima* in particular, are biogeographically widespread. The species occurs from the high Arctic to the cold-temperate region of the North Atlantic (Lüning 1990). Müller et al. (2008) have demonstrated ecotypic differentiation with respect to interactive effects of UV radiation and temperature on microstages of various kelps including *S. latissima* from the Arctic and the North Sea. Hence, it is reasonable to hypothesize that the Arctic ecotype is adapted to low temperatures and relatively high [CO_2_] dissolved in seawater, although the prevailing [CO_2_] within dense kelp forests can be very low due to the high photosynthetic activity of brown algae as demonstrated in sub-Antarctic/cold-temperate waters (Delille et al. 2009). Consequently, the biochemical composition (BC) (e.g., content of C, N, C/N-ratio, proteins, carbohydrates, and lipids) of polar and temperate populations of this species might be generally different even if the algae are cultured under equal standardized conditions, meaning that differences are genetically programmed.

Generally, very little is known about the change in the biochemical composition of kelp under changing environmental conditions such as a rise in temperature and a lowering of the pH of seawater due to globally occurring climatic changes (Müller et al. 2009; Barry et al. 2010). During acclimation to changing temperatures, the metabolism is adjusted (Davison 1991) and, consequently, the BC of kelps is certainly affected. Clearly, seasonality also affects the BC of mature kelp sporophytes and zoospores (Black 1948; Hernández-Carmona et al. 2009; Adams et al. 2011; Olischläger and Wiencke 2013a). The amount of soluble carbohydrates in kelp is clearly affected by seasonality, with highest values of most carbohydrates (except alginic acid) reported for the summer months (Black 1948; Hernández-Carmona et al. 2009; Adams et al. 2011; Westermeier et al. 2012). In *S. latissima*, the protein content decreases in parallel (Black 1948). Highest values for total lipids were found in winter as shown in several species of marine macroalgae (Nelson et al. 2002). However, the precise contribution of temperature itself on the seasonal differences is less clear since seasonal differences in the BC might be strongly influenced by further environmental factors such as light regime, nutrient availability, and the particular life strategy of the species (Bartsch et al. 2008).

The protein content, the N-content, and the C/N-ratio can be affected by temperature since temperature affects the activity of enzymes, and these changes in activity can be counterbalanced by a change in the amount of protein (Raven and Geider 1988; Davison 1991). For microalgae, decreased protein or N-contents as response to elevated temperatures are often reported (e.g., Thompson 1999; Renaud et al. 2002; Carvalho et al. 2009), a change which can be, but not necessarily has to be, accompanied by an increase in C-storage compounds such as lipids and carbohydrates (Carvalho et al. 2009). Nevertheless elevated temperatures can also have no significant effect on the protein content and still lead to pronounced changes in lipid and carbohydrate content (de Castro Araújo and Tarvano Garcia 2005; Gigova et al. 2012). The heterogeneity of the findings is likely to be explained by species specificity, but also by the known strong interactive effects between temperature and other factors such as nutrients, light, and day length (Thompson 1999; Carvalho et al. 2009).

Raven et al. (2002) hypothesized that the impact of low temperatures on photosynthesis by marine macrophytes favors diffusive CO_2_ entry rather than stimulating the CO_2_-concentrating mechanism. However, Gordillo et al. (2006) measured high activities of HCO_3_^−^ utilizing enzymes in polar macroalgae collected from the field. Since at low temperatures enzyme activities and the diffusion coefficients of CO_2_ decrease (Raven and Geider 1988; Raven et al. 2002), Gordillo et al. (2006) explained the particularly high expression of these enzymes as part of the acclimation strategy to the cold Arctic environment, counteracting the unbalance between the photochemical reactions, which are temperature independent, and the enzymatically driven reactions of the Calvin cycle, which are temperature dependent, thus preventing photoinhibition.

Moreover, the BC of macroalgae can be affected by the availability of dissolved CO_2_ (e.g., Andría et al. 2001; Gordillo et al. 2001a, b; Swanson and Fox 2007) and within the Laminariales life cycle the photosynthetically active stages are known to be sensitive to elevated pCO_2_ (Olischläger et al. 2012). A high pCO_2_ can cause the downregulation of enzymes involved in carbon assimilation (Giordano et al. 2005), which in turn can lower the algal protein, and/or N-content in some red and green macroalgae species (Andría et al. 2001; Gordillo et al. 2001a, b). Under nutrient replete conditions, these changes can be reflected in a higher C/N-ratio and can be accompanied by insignificant changes in the contents of soluble carbohydrates and lipids (Gordillo et al. 2001b). In contrast, in marine phytoplankton elevated pCO_2_ can increase the content of proteins in parallel to unchanged contents of carbohydrates and lipids (Brown et al. 1997) or decreased amounts of carbohydrates (de Castro Araújo and Tarvano Garcia 2005). Also, the elemental composition of several marine phytoplankton species was shown to be dependent on the prevailing pCO_2_, but generalizations with respect to the direction of C/N-ratio under predicted pCO_2_ were not possible (Burkhardt et al. 1999). Conclusively, the response of marine algae to changing pCO_2_ appears to be species specific, but it has to be pointed out that all mentioned studies were performed with microalgae or red and green macroalgae being phylogenetically or in terms of habitat and life strategies fairly different from kelp. Furthermore, except the study of Burkhardt et al. (1999), the experimental approaches of most previous studies cannot be considered as adequate for the prediction of elevated pCO_2_ on marine photoautotrophs, since buffered media and/or unrealistically high pCO_2_ were applied (e.g., Brown et al. 1997; Andría et al. 2001; Gordillo et al. 2001a). It became established that buffered media can strongly inhibit the carbon concentrating mechanism (CCM) of red and brown algae (e.g., Mercado et al. 2006; Moulin et al. 2011), and studies performed with buffered seawater or very high pCO_2_, and, respectively, low pH, are of limited usefulness for the prediction of climate change effects on kelp.

In the present paper, we tested the hypotheses whether (1) there is an ecotypic variation in the chemical composition of Arctic and temperate populations of *S. latissima*, (2) temperature and increased pCO_2_, separately or interactively, affect the BC of *S. latissima*, and (3) the responses of Arctic and temperate populations of *S. latissima* are different to changing pCO_2_ with correspondingly low pH and temperature.

## Materials and methods

### Algal material and experimental conditions

Young vegetative sporophytes of *S. latissima* Linnaeus were raised from gametophytes kept in AWI-stock cultures isolated from Helgoland (HL), North Sea (AWI-culture number: ♂-gametophytes 3,094, ♀-gametophytes 3,096) and Spitsbergen (SP), Arctic (AWI-culture number: ♂-gametophytes 3,123, ♀-gametophytes 3,124). Male and female gametophytes from the two populations were mixed separately and carefully fragmented with pestle and mortar. The developing sporophytes were kept in dim white light (15–20 µmol photons m^−2 ^s^−1^) at 10 °C until experimental use. As light source, we used fluorescent tubes (Osram 58 W/965 Biolux, Munich, Germany) throughout the study. The photon fluence rate (PFR) was adjusted to 70 ± 10 µmol photons m^−2 ^s^−1^ at the bottom and 120 ± 10 µmol photons m^−2 ^s^−1^ at the top of the beaker. PFRs were measured using a flat-head cosine-corrected quantum sensor attached to a radiometer (Li-185-B, flat-head quantum sensor; LI-COR Biosciences, Lincoln, NE, USA).

For the experiments 0.5 ± 0.1 g fresh weight of algae were transferred to 5 L beakers filled with filtered seawater (FSW; 0.2 µm), enriched with unbuffered nutrients after Provasoli (1968) including 2.0 mM NO_3_^−^ and 0.05 mM PO_4_^2−^, and aerated continuously with artificial air (20 % oxygen, 80 % nitrogen) with a target pCO_2_ of 380, 800, or 1,500 µatm generated by a gas mixing device (HTK GmbH, Hamburg, Germany). Further on, these pCO_2_ treatments are called present, expected, and high pCO_2_. FSW was aerated with the different gas mixtures described above for 24 h prior to experimental use. FSW was exchanged every 3–4 days. Thalli were moved continuously by aeration and cultivated under described conditions for 18 days in temperature-controlled rooms adjusted to 17 °C ± 1.5 °C and 10 °C ± 1.5 °C for the Helgoland population and 10 °C ± 1.5 °C and 4 °C ± 1.5 °C for the Spitsbergen population.

### Monitoring of the seawater carbonate system during the experiment

The seawater carbonate system (SWCS), including the pCO_2_ of the FSW was monitored by taking 250 ml samples in the beginning of the experiment and every 3–4 days throughout the entire experimental period. Temperature in the beakers was controlled using a submersible thermometer (WTW-LF 197-S, WTW-GmbH, Weilheim, Germany). pH, electromotive force (mV) and salinity were measured at 25.0 ± 0.1 °C (pH: Ioline-electrode; SI Analytics GmbH, Mainz, Germany, attached to a WTW-720 pH-meter; salinity: WTW-LF 197-S, WTW-GmbH). As recommended by Dickson et al. (2007), the pH was expressed on a total scale. The pH T was calculated according to Dickson et al. (2007) from the electromotive force of the seawater sample and the electromotive force and pH of Tris-buffer seawater standards (Oceanic Carbon Dioxide Control, Scripps Institution of Oceanography, San Diego, CA, USA) using Eq. 1.

1 $${\text{pH }}T\; = \;{\text{pH(}}S )+ \frac{E (s )- E (x )}{{RT\;{\text{ln(}}10/F )}}$$

Equation 1 where pH (*T*) = pH of the sample on the total scale, pH (*S*) = pH of the seawater standard, E(*s*) = electromotive force of the seawater standard, E(*x*) = electromotive force of the seawater sample, *R* = gas constant, *T* = Temperature in K, *F* = Faraday constant.

Alkalinity total (AT) was determined by automatic potentiometric titration of 25 mL of seawater medium with 0.05 M HCl containing 35 g L^−1^ NaCl by use of an automated titration system (TW-alpha plus, SI Analytics, Mainz, Germany) and calculated from linear gram plots (Gran 1952). The components of the marine carbonate system were calculated with CO2SYS software (Lewis and Wallace 1998) using the equilibrium constants for the dissociation of carbonic acid in seawater from Millero et al. (2006), and for sulfuric acid the constants of Dickson (1990). However, for one measuring date in SP 10 °C-treatment, the pH was measured on the National Bureau of Standards (NBS)-Scale due to a technical failure. The SWCS calculations of this measurement date used the dissociation constants for carbonic acid from Takahashi et al. (1982), which are recommended for the NBS-scale. Detailed values of the measured characteristics of the SWCS are presented in Table 1.

**Table 1:** Characteristics of the seawater carbonate system (mean + standard deviation) in the different experiments over the entire experimental period

Experiment	pH	TA µmol kg SW^−1^	Temp. °C	pCO_2_ µatm	HCO_3_^−^ µmol kg SW^−1^	CO_3_^−^ µmol kg SW^−1^	TC µmol kg SW^−1^
HL 17 °C present pCO_2_	8.06 ± 0.03	2,426 ± 25	17.0 ± 0.3	421 ± 38	2,000 ± 36	175 ± 12	2,190 ± 31
HL 17 °C expected future pCO_2_	7.82 ± 0.03	2,422 ± 18	16.8 ± 0.4	783 ± 65	2,158 ± 27	108 ± 8	2,294 ± 24
HL 17 °C high pCO_2_	7.56 ± 0.02	2,422 ± 12	17.1 ± 0.4	1,505 ± 83	2,268 ± 12	63 ± 4	2,384 ± 12
HL 10 °C present pCO_2_	8.06 ± 0.02	2,421 ± 46	9.7 ± 0.1	423 ± 20	2,094 ± 35	134 ± 9	2,247 ± 39
HL 10 °C expected future pCO_2_	7.82 ± 0.02	2,397 ± 27	10.1 ± 0.2	769 ± 56	2,196 ± 23	82 ± 5	2,313 ± 13
HL 10 °C High pCO_2_	7.56 ± 0.02	2,401 ± 24	10.1 ± 0.1	1,437 ± 51	2,285 ± 21	48 ± 2	2,396 ± 22
SP 10 °C present pCO_2_	8.07 ± 0.02	2,412 ± 29	9.7 ± 0.2	433 ± 51	2,102 ± 40	128 ± 12	2,249 ± 34
SP 10 °C expected future pCO_2_	7.89 ± 0.03	2,411 ± 30	9.9 ± 0.2	683 ± 58	2,195 ± 28	89 ± 8	2,314 ± 38
SP 10 °C high pCO_2_	7.58 ± 0.02	2,405 ± 26	9.9 ± 0.2	1,456 ± 107	2,295 ± 23	45 ± 4	2,406 ± 25
SP 4 °C present pCO_2_	8.07 ± 0.03	2,391 ± 42	3.8 ± 0.2	402 ± 27	2,125 ± 45	108 ± 7	2,255 ± 45
SP 4 °C expected future pCO_2_	7.88 ± 0.05	2,381 ± 19	4.3 ± 0.2	644 ± 89	2,198 ± 33	75 ± 9	2,308 ± 30
SP 4 °C high pCO_2_	7.57 ± 0.04	2,386 ± 20	4.2 ± 0.2	1,354 ± 111	2,292 ± 22	38 ± 3	2,404 ± 24

*HL* Helgoland population (gray), *SP* Spitsbergen population (white)

### Biochemical composition

Fresh algal material was taken from the beaker, rinsed with Milli-Q-water to remove salt, dried with tissue paper, weighed, and frozen in liquid nitrogen within minutes. Samples for BC were freeze dried, ground in a Mixer Mill (MM 400, Retsch) and the dry weight determined. Subsamples of the homogenates were analyzed for their total lipids, total proteins, and soluble carbohydrate content.

### C/N-ratios

Samples were milled and exposed to HCl-vapor for 4 h at room temperature in an extraction chamber to remove inorganic C and then milled again. Concentration measurements of nitrogen and carbon were performed simultaneously with a Thermo/Finnigan MAT V isotope ratio mass spectrometer, coupled to a Thermo Flash EA 1112 elemental analyzer via a Thermo/Finnigan Conflo III interface.

### Carbohydrates

Soluble carbohydrates were extracted from freeze-dried material in distilled water at 80 °C for 2 h, and quantified by phenol–sulfuric acid method (Kochert 1978), using glucose as standard. Carbohydrates are then expressed as glucose equivalents.

### Proteins

Total protein extraction procedure was modified from the method described by Kim et al. (2011). Fifty mg of freeze-dried material was homogenized in 1 mL of 0.1 M MOPS (pH 7), 7 M urea, 4 % SDS, 2 M thio-urea, 100 mM DTT, 2 mM EDTA, 4 % PVP-40, 1 mM PMSF, 1 mM ε-amino-*n*-caproic acid and 10 μM leupeptin. Samples were centrifuged (14,000*g*, 30 min, 4 °C). The resultant supernatants were recovered, and the pellets containing debris were removed. To eliminate interfering compounds, proteins were precipitated by the addition of an equal volume of 20 % trichloroacetic acid in acetone at −20 °C overnight. After centrifugation (14,000*g* for 30 min, 4 °C), the supernatant was discarded and the pellet washed two times with 1 mL acetone pre-chilled to −20 °C. The pellet remaining after the second wash was allowed to dry at 4 °C, and was resuspended in 200 μL of 4 % SDS. Protein concentration was determined by the BCA assay (Smith et al. 1985), using bovine serum albumin as standard.

### Lipids

Total lipids were extracted from freeze-dried material in 2:1 (v/v) chloroform–methanol mixture and quantified by the sulfo-phospho-vanillin method (Barnes and Blackstock 1973) using cholesterol as standard.

### Statistics

Homogeneity of variances was confirmed using the Levene’s test (*P* < 0.05). Two-factorial designs were analyzed with a two-way-ANOVA (*P* < 0.05). If homogeneity of variances could not be achieved, the two-way-ANOVA was performed with a reduced p-level of *P* < 0.01 to counteract the increased risk of an α-error. First, we tested for each chemical component the influence of populations and pCO_2_ in a two-factorial design. In this assay, we examined the chemical composition measured at 10 °C. 10 °C was chosen for the comparison of both populations, since the 10 °C August isotherm is considered to be the border of the Arctic but it is also a frequently occurring temperature in the cold-temperate environment of the North Sea (Lüning 1990). Accordingly, at the northern continental Norwegian coast both ecotypes could coexist (Lüning 1990). Secondly, we tested the influence of pCO_2_ and temperature for each population. For this assay, the chemical composition from 17 °C and 10 °C were measured for the HL-population and at 10 °C and 4 °C for the SP-population. Post hoc comparisons were performed by Fisher’s LSD test. The analyses were performed using Statistica software v.7 (StatSoft Inc, Tulsa, OK, USA).

## Results

### The effect of pCO_2_ and temperature on the chemical composition of the Helgoland population

**Fig. 1:**
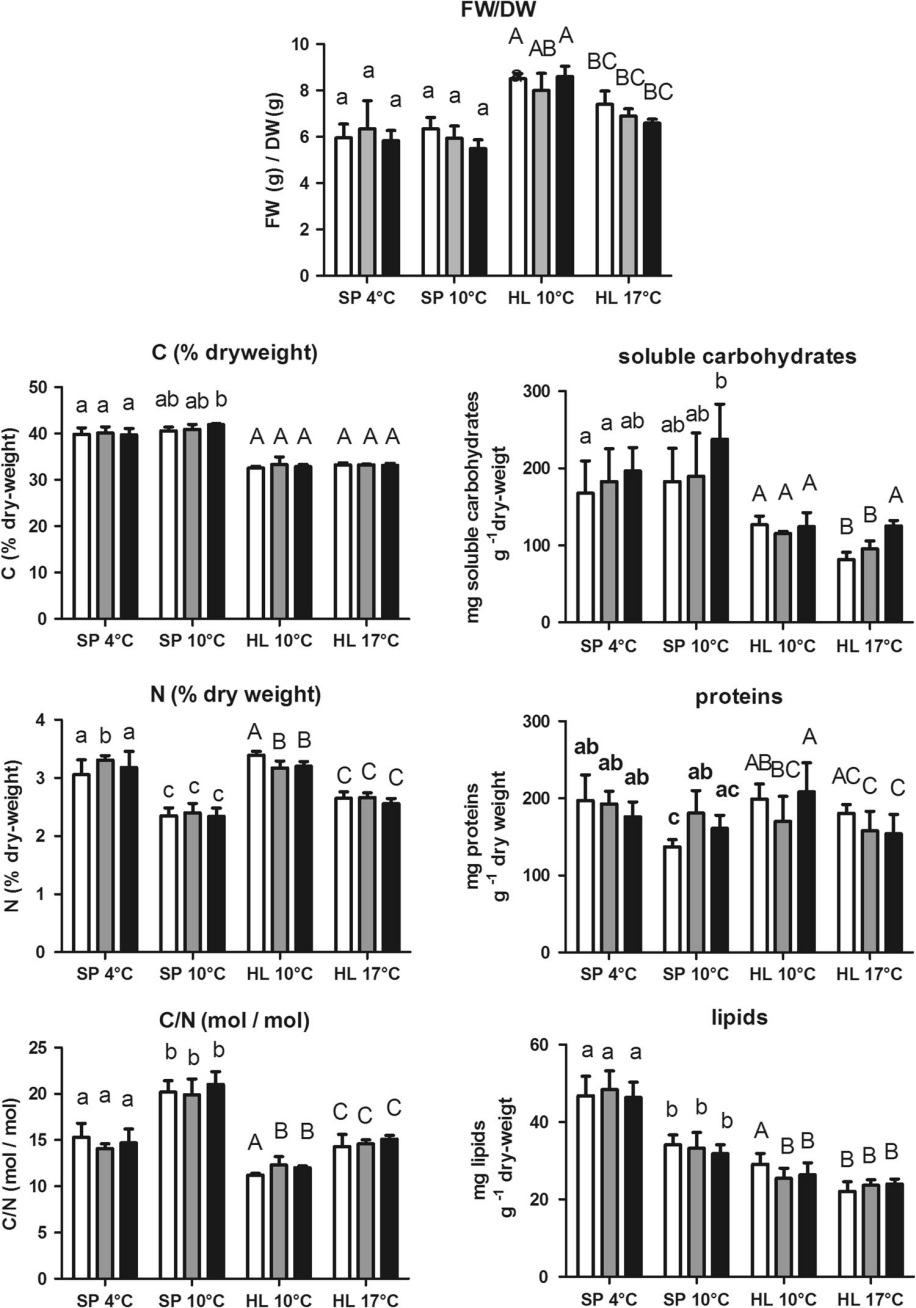
Chemical components (mean ± standard deviation) of *Saccharina latissima* from Helgoland (HL) or Spitsbergen (SP), cultivated at indicated temperatures and at present pCO_2_ (*white bar*), expected future pCO_2_ (*gray bar*) or high pCO_2_ (*black bar*). Significant differences, revealed by Fisher’s LSD test, following a two-factorial ANOVA (pCO_2_ and temperature) performed with the HL- or SP-population are indicated by *small letters* (SP-population) or *capital letters* (HL-population)

Except the total C-content all tested chemical components were significantly affected by temperature (*P* < 0.01; two-factorial ANOVA). The contents of soluble carbohydrates, lipids, proteins, N, and the FW/DW-ratios were significantly higher at 10 °C than at 17 °C for the HL-population (*P* < 0.01; two-factorial ANOVA), whereas the C/N-ratio was significantly lower at 10 °C (*P* < 0.01; two-factorial ANOVA; Fig. 1; Table 2).

**Table 2:** Results of the testing for significant influences of temperature, cultivation-pCO_2_, and the interaction of temperature and cultivation-pCO_2_ on the chemical composition of *Saccharina latissima* from temperate latitudes (Helgoland = HL, gray) and Spitsbergen (SP, white) by a two-way ANOVA

Chemical characteristic	Temperature	pCO_2_	Temperature *pCO_2_
Fresh weight/dry weight (HL)	***	n.s.	n.s.
Fresh weight/dry weight (SP)	n.s.	n.s.	n.s.
C (% dry weight) (HL)	n.s.	n.s.	n.s.
C (% dry weight) (SP)	***	n.s.	n.s.
N (% dry weight) (HL)	***	***	*
N (% dry weight) (SP)	***	n.s	n.s.
C/N (mol mol^−1^) (HL)	***	*	*
C/N (mol mol^−1^) (SP)	***	n.s.	n.s.
Lipids (mg g^−1^ dry weight) (HL)	***	n.s	*
Lipids (mg g^−^1 dry weight) (SP)	***	n.s.	n.s.
Proteins (mg g^−1^ dry weight) (HL)	***	n.s.	n.s.
Proteins (mg g^−1^ dry weight) (SP)	***	n.s.	*
Soluble carbohydrates (mg g^−1^ dry weight) (HL)	***	***	***
Soluble carbohydrates (mg g^−1^ dry weight) (SP)	n.s.	n.s.	n.s.

* *P*-level < 0.05; *** *P*-level < 0.01

Expected and high pCO_2_ significantly lowered the algal N-content (*P* < 0.05; two-factorial ANOVA) but only at 10 °C (*P* < 0.01; Fisher’s LSD test), whereas at 17 °C the pCO_2_ had no influence on the N-content (*P* > 0.05; Fisher’s LSD test). Accordingly, significant interaction of pCO_2_ and temperature on the N-content became evident (*P* < 0.05; two-factorial ANOVA). Likewise, the C/N-ratio at present pCO_2_ and 10 °C was significantly lower compared to the C/N-ratio measured at expected future and high pCO_2_ (*P* < 0.05; Fisher’s LSD test). At 17 °C, no pCO_2_ specific difference was found (*P* > 0.05; Fisher’s LSD test). Again, the interaction of temperature and pCO_2_ on the C/N-ratio was significant (*P* < 0.05; two-factorial ANOVA). In contrast, the protein content and the FW/DW-ratio were not significantly affected neither by pCO_2_ nor by the interaction of temperature and pCO_2_ (*P* > 0.01; two-factorial ANOVA). The content of soluble carbohydrates was significantly affected by pCO_2_ and was temperature dependent (*P* < 0.01; two-factorial ANOVA). It increased at high pCO_2_ and 17 °C, whereas at 10 °C pCO_2_ had no effect (*P* > 0.05, Fisher’s LSD test). The effect of pCO_2_ on the lipid content alone was not significant (*P* > 0.05; two-factorial ANOVA). On the other hand, at 10 °C the thalli cultivated at present pCO_2_ contained significantly more lipids than in material cultivated at expected and high pCO_2_ (*P* < 0.05; Fisher’s LSD test). In contrast, at 17 °C, the lipid content was not significantly affected by pCO_2_ treatments (*P* > 0.05; Fisher’s LSD test). Accordingly, the pCO_2_ effect was depended on temperature and both factors were interactive (*P* < 0.05; two-factorial ANOVA).

### The effect of pCO_2_ and temperature on the chemical composition of the Spitsbergen population

Except for the content of soluble carbohydrates and the FW/DW-ratio, temperature affected all tested chemical components significantly (*P* < 0.01; two-factorial ANOVA). The content of lipids, proteins, and total N-content increased significantly at 4 °C compared to 10 °C (*P* < 0.01; two-factorial ANOVA), whereas the content of total C decreased slightly but significantly (*P* < 0.01; two-factorial ANOVA). The C/N-ratio was also significantly lower at 4 °C (*P* < 0.01; two-factorial ANOVA, Table 2).

Elevated pCO_2_ alone did not significantly affect any of the tested chemical components (*P* > 0.05; two-factorial ANOVA). However, elevated pCO_2_ and temperature influenced interactively the total protein content of the thalli (*P* < 0.01; two-factorial ANOVA). Expected and high pCO_2_ could significantly counteract the increase in the total protein content caused by lower temperature. Protein content was only significantly higher at 4 °C and at present pCO_2_ relative to that at 10 °C (*P* < 0.05; Fisher’s LSD test), whereas no significant difference in the protein content between the 4 °C and 10 °C-treatment was found at expected and high pCO_2_ (*P* > 0.05; Fisher’s LSD test).

### Ecotypic variation and the effect of pCO_2_ on the chemical composition between the two populations studied

The examined independent factors analyzed here were ecotype and pCO_2_ at 10 °C. In this arrangement, the SP-population had a significantly higher content of C, soluble carbohydrates and lipids and a higher C/N-ratio, but a lower content of N and proteins, and FW-DW-ratio than the HL-population (*P* < 0.01; two-factorial ANOVA; Fig. 1; Table 3). pCO_2_ alone had no significant effect on any of the tested chemical components at 10 °C (*P* > 0.05; two-factorial ANOVA; Table 2) but a significant interaction of pCO_2_ and the ecotype was evidenced in the N- and protein content (*P* < 0.05; respectively, *P* < 0.01; Table 3).

**Table 3:** Results of testing for significant differences in the chemical composition by Arctic and temperate populations of *Saccharina latissima* (ecotypes) at 10 °C, the influence of cultivation pCO_2_ on the chemical composition of different populations, and the interaction of cultivation pCO_2_ and ecotype by two-way-ANOVA

Parameter	Ecotype	pCO_2_	Ecotype *pCO_2_
Fresh weight/dry weight	***	n.s.	n.s.
C/N	***	n.s.	n.s.
C (% dry weight)	***	n.s.	n.s.
N (% dry weight)	***	n.s.	*
Lipids (mg % g^−1^ dry weight)	***	n.s.	n.s.
Proteins (mg % g^−1^ dry weight)	***	n.s.	*
Soluble carbohydrates (mg % g^−1^ dry weight)	***	n.s.	n.s.

* *P*-level < 0.05; *** *P*-level < 0.01

## Discussion

### The biochemical composition of *S. latissima*

The values obtained in this study for fresh weight (FW)/dry weight (DW)-ratio for Arctic *S. latissima* are similar to previously reported field values (Gordillo et al. 2006). The lower content of water in the SP-population might be related to the osmolyte concentration as a genetic adaptation to cold environments, since one acclimation strategy under low temperatures seems to be the increase of soluble cell components (Raven and Geider 1988; Davison 1991). Hence, the FW/DW-ratios support Davison and Davison (1987), who postulated that in *S. latissima* the concentrations of osmolytes, such as NO_3_^−^, amino-acids, and mannitol increase at low temperatures. Likewise, the measured C- and N-content of the HL-population are similar to values reported for specimens collected in the English Channel (Gevaert et al. 2001), whereas the SP-population has comparable N-values but higher C-values than the temperate one. However, the C-content of both populations is higher than that reported for *S. latissima* field thalli from British Columbia, while the N-content of the British Columbia population is lower than the N-content of the populations tested in this experiment (Ahn et al. 1998).

The measured C- and N-contents of the SP-thalli are much higher than values for Arctic field grown thalli (Gordillo et al. 2006) incubated in nutrient-enriched seawater. This deviation might be due to their particular experimental conditions. Gordillo et al. (2006) used summer field material exposed to low nutrient conditions and 24 h of sunlight during the polar day, including UV-exposure for several months. Temperate *S. latissima* can store N internally, but these internal N-reserves are depleted after 3 months under low external N-supply (Korb and Gerard 2000), hence, the low N-content of Arctic field thalli shown in Gordillo et al. (2006) could be explained by an ongoing depletion of internally stored nitrogen during the course of the nutrient poor polar summer. Furthermore, beside the nutrient availability, the day length and the radiation regime might affect the C-content.

A further reason might be the high content of soluble carbohydrates. The sugar alcohol mannitol is one of the main photosynthetic products, and serves as a storage compound along with the polysaccharide laminaran in brown algae (Bartsch et al. 2008). Arctic brown macroalgae accumulate C-storage molecules during summer that support new tissue growth during the following dark winter (Dunton and Schell 1986). Hence, the high soluble carbohydrate content in the SP-population could be due to the accumulation of C-storage molecules in light, despite the replete nutrient concentration in the medium, as a consequence of a seasonal developmental strategy of Arctic species. The latter would also help to explain the lower growth rate of the SP-population compared to HL-population (Olischläger et al. unpublished data). Generally, the content of carbohydrates in *S. latissima* is much higher than in *Macrocystis pyrifera* (Westermeier et al. 2012). The content of carbohydrates determined for SP-population in the present study is similar to that obtained for the brown tropical alga *Sargassum filipendula* (Diniz et al. 2011), although comparing with HL-population, *S. filipendula* is characterized by a higher carbohydrate content. The contents of total lipids in HL-population and *S. filipendula* are similar. The much higher content of lipids of *S. latissima*, particularly under cold conditions, compared to field values reported for other kelp species (Hernández-Carmona et al. 2009; Westermeier et al. 2012) is also remarkable. As discussed below, this could also be a part of the adaptation to extremely low temperature.

Protein content is much higher in the present study than in field thalli (Gordillo et al. 2006), mainly because we measured total proteins whereas they estimated only soluble proteins. However, the difference in the protein content between studies might also be related to differences in the effectiveness of different protein extraction procedures used (Iñiguez et al. unpublished data), and also in the spectrophotometric method used, as protein contents determined by use of the Bradford method results in values 25–50 % lower than obtained by use of the bicinchoninic acid method (Berges et al. 1993). The fact that this is the first time that a kelp-specific extraction method is used rendering high efficiency explains why the protein content from both temperate and Arctic *S. latissima* (Fig. 1) was higher than those reported for most other brown algae except *Undaria pinnatifida* (Fleurence 1999; Hernández-Carmona et al. 2009; Westermeier et al. 2012), and similar to values obtained in the red alga *Hypnea spinella* (Suárez-Álvarez et al. 2012).

The C/N-values of Arctic *S. latissima* from the field and from the laboratory are high compared to temperate populations (Gordillo et al. 2006; present study, Fig. 1), whereas the C/N-values reported for temperate field thalli from *S. latissima* (Ahn et al. 1998; Gevaert et al. 2001) are comparable to the values obtained in the present laboratory study at 10 °C. High C/N-values in Arctic-adapted populations are in agreement with a C-accumulating summer metabolism as earlier mentioned.

### Ecotypic differentiation and the effect of pCO_2_ on the biochemical composition

An important question in terms of species responses to environmental change is whether variation in life-history traits between populations are based on acclimation through phenotypic plasticity (no genetic change) and/or through physiological adaptation with the development of genetically distinct ecotypes (Lobban and Harrison 1997; Pigliucci et al. 2006). Phenotypic plasticity expands the ecological range of a species, thereby exposing it to new selective pressures, allowing for genetic adaptation when exposed for sufficient time periods (Pigliucci et al. 2006; Nicotra et al. 2010). Two genetically different ecotypes express a diversified trait for a given environmental condition (Spurkland and Iken 2012) Thus, different chemical composition (involving all the chemical components analyzed here) under the same environmental conditions (at 10 °C) for SP- and HL-populations (Fig. 1, Table 3) indicate that both populations represent different ecotypes. This is further supported by the different response pattern to changing pCO_2_. In addition, ecotypic differentiation could also be observed in the different sensitivity of developmental stages of this and other kelp species to UV radiation and temperature (Müller et al. 2008). For a given temperature, we know that populations also differ in photosynthesis and growth rates (Olischläger et al. unpublished data). Hence, our results support that SP- and HL-populations are different ecotypes, although a genetic confirmation would be desirable.

If the effects of elevated pCO_2_ are compared between the two populations at 10 °C, elevated pCO_2_ does not result to influence the BC significantly. However, a significant interaction between CO_2_ and ecotype was revealed for N- and protein content.

### The impact of temperature and pCO_2_ on the chemical composition of the two *S. latissima* populations

Most of the chemical components measured were significantly affected by temperature in both ecotypes but they were not affected by pCO_2_, indicating that *S. latissima* was more sensitive to changes in water temperature than to changes in the concentration of dissolved CO_2_ in the range used here. We also observed that the response to pCO_2_ was ecotype-specific, with a rather pCO_2_-insensitive Arctic population and a significantly sensitive temperate population. This insensitiveness to pCO_2_ of the Arctic population could be the result of an adaptation to low temperature. At low temperatures, the uncoupling between C-fixation (temperature dependent) and the photochemical reactions (temperature independent) make cells prone to photoinhibition. It has been suggested that polar algae might maintain CCMs constitutively active (rather than being repressed at high CO_2_ as their temperate counterparts), promoting high CO_2_ fixation rates independent of pCO_2_ as a photoprotective mechanism that allow for a functional and effective C-fixation (Gordillo et al. unpublished results).

Both populations exhibit a lower total N- and higher C/N-ratio at the tested high temperature. This behavior of the C/N-ratio is in agreement with Gevaert et al. (2001) who showed higher C/N-values in summer in *S. latissima* thalli from the English Channel, and also Dunton and Schell (1986), who showed the same behavior in the Arctic species *Laminaria solidungula*. Hence, it is reasonable to conclude that moderately elevated temperatures are leading to higher C/N-ratios in *S. latissima* under both field and laboratory conditions.

The higher N-content at low temperatures might be partly attributed to the enzyme quantity. A higher amount of enzymes is needed to achieve the same catalytic activity at low temperatures (Davison 1991; Young et al. 2007). However, the temperature effect on the total N-content is more pronounced than the changes in the protein content. This finding might be explained in two ways. Firstly, the temperature optima of N-acquiring enzymes in *S. latissima* are between 7 °C and 10 °C (Davison and Davison 1987; Young et al. 2007), a fact which might be reflected in the low total N-content at the higher tested temperatures. Secondly, it was previously suggested that in kelp acclimatization to low temperatures requires an accumulation of osmolytes, and that NO_3_^−^ is among these osmolytes (Davison and Davison 1987; Bartsch et al. 2008), by this way contributing to the measured higher N-content at low temperatures.

Changes in total C are not completely explained by changes in soluble carbohydrates, so that temperature influences the type of energy rich molecule accumulated—either carbohydrates (at high temperatures) or lipids (at low temperatures). Whereas in SP-thalli, the total C-content decreased at low temperatures, temperature had no significant effect on the C-content of the HL-thalli. Nevertheless, the C-content of the SP-thalli is generally higher compared to the HL-thalli and the magnitude of the temperature effect on the C-content of the SP-thalli is rather small (~2–5 %), despite the proven significance. Within the SP-population, the amount of soluble carbohydrates did not increase at lower temperatures but the lipid content did, whereas carbohydrates increased in the HL-thalli. This finding could be due to an increase in the fraction of photosynthates being stored as lipids serving as a reserve for growth during the polar night.

Lipids were higher in both populations at low temperatures but the effect is more pronounced in the Arctic population. It was previously shown in the green phylogenetically distinct macroalga *Ulva* *pertusa* that the content of lipids increases at low temperatures (Floreto et al. 1993), and also during the winter months in the kelp *Eisenia arborea* (Hernández-Carmona et al. 2009). An increase in lipid content as a response to low temperature has been widely observed; however, as far as we know, this is the first report on an increased content of lipids in a polar seaweed relative to their cold-temperate counterpart. The generally higher lipid content of the Arctic population could indicate a cold adaptation because lipids are regarded as a more energetic storage compound (Nelson and Cox 2002). Increasing the proportion of energy stored in form of lipids than in carbohydrates increases the total amount of stored energy, since the amount of energy stored per bound C in lipids is roughly twice times more than the amount of energy stored per bound C in carbohydrates (Nelson and Cox 2002). However, for the formation of storage lipids two acetyl-CoA (each with two C-atoms) are successively incorporated. These acetyl-CoA derive from pyruvates, which in turn originate from in the photosynthetic dark reaction produced hexoses (Sitte et al. 2002). Consequently, more than one-third of the in the hexose stored energy is lost if storage lipids are produced. This high energetic investment for the formation of lipids in the SP-thalli is explainable, if the particular environmental circumstances, which Arctic algae face, are considered. During the polar day, when polar *S. latissima* encounters 24 h of sunlight, the alga can effectively photosynthesize due to high nutrient availability in spring and internally stored nutrients in summer (Lüning 1990; Korb and Gerard 2000). At this time of nutrient abundance, the algae can afford to invest a considerable amount of energy in the production of lipids, which are, in terms of stored energy per volume unit, the more effective energy storage metabolite. This might be needed to survive the months lasting polar night. Accordingly, our data indicate that the selective pressure to develop an more effective energy storage is more pronounced at high latitudes. It has also been shown that the accumulation of polyunsaturated fatty acids in the biological membranes is an adaptive mechanism to cold environments allowing for the maintenance of membrane fluidity (Morgan-Kiss et al. 2006).

Furthermore, the cold-acclimation can be facilitated via an increase in the functional protein content (Raven and Geider 1988; Davison 1991) that counteracts the decline of catalytic activity at low temperatures, and kelp is known to have higher protein content in winter (Black 1948; Hernández-Carmona et al. 2009; Westermeier et al. 2012). Our study confirms that, the total protein content significantly increased at low temperature, in agreement with the mentioned acclimation strategy (Raven and Geider 1988; Davison 1991).

In the HL-population, elevated pCO_2_ lowers the N-content and causes a higher C/N-ratio but only if the thalli are cultured at 10 °C, while in the SP-population the N-content was not affected by pCO_2_. In this sense, Olabarria et al. (2012) showed that the N-content of the red, respectively, brown seaweed *Chondrus crispus* and *Cystoseira tamariscifolia* were positively affected by an increase in pCO_2_, while the red alga *Mastocarpus stellatus* and the brown seaweeds *Sargassum muticum* were not affected. On the other hand, the red alga *Hypnea spinella* responded in a similar way than HL-population at 10 °C, increasing C/N-ratio at elevated pCO_2_ conditions, while C-content remain constant (Suárez-Álvarez et al. 2012). Experimental studies have shown that responses of internal N-content to CO_2_ enrichment vary greatly between different algae (Gordillo et al. 1999; Andría et al. 2001). This decrease in the N-content at 10 °C and elevated pCO_2_, which is not reflected in a protein content change, could be due to a lower accumulation of inorganic N inside the cell, as brown algae accumulate around 22 % of non-protein N (Diniz et al. 2011), and *S. latissima* has been shown to possess considerable NO_3_^−^ pools (Korb and Gerard 2000). However, the physiological reason for the potential decrease in the NO_3_^−^ pool remains unclear.

An increase in the C/N-ratio of similar magnitude of the one found in our study in the HL-population at 10 °C was recently reported for diatoms after cultivation at elevated pCO_2_, combined with a lower transcription of a δ-carbonic anhydrase (Crawfurd et al. 2011). However, in *Saccharina japonica* pH-changes affected many metabolic pathways beside carbon acquisition (Kim et al. 2011). For Arctic algae, adapted to cold- and high CO_2_-concentrations, Raven et al. (2002) postulated that the high concentration of dissolved CO_2_ in cold Arctic waters would decrease the need to express a CCM. This could be reflected in the shown insensitivity of the C/N-ratio and the N- and protein content of the SP-population to elevated pCO_2_.

In marine plants and macroalgae, cultivation under controlled conditions and elevated pCO_2_ revealed species-specific results. The content of carbohydrates has been shown to increase as response to elevated pCO_2_ in the seagrass *Thalassia hemprichii* (Jiang et al. 2010) and in *H. spinella* (Suárez-Álvarez et al. 2012), whereas the carbohydrate content of *U. rigida* under N-replete conditions was not affected by elevated pCO_2_ (Gordillo et al. 2001a). We could show that, under replete nutrient concentration, the effect of elevated pCO_2_ on the carbohydrate content of *S. latissima* is ecotype specific, with an insensitive Arctic population and a pCO_2_-sensitive temperate population. The temperate population accumulates more carbohydrates at elevated pCO_2_, but only if the algae are cultured at high temperatures. Conclusively, the carbohydrate content of warm water ecotypes appears to be more influenced by elevated pCO_2_.

We showed that pCO_2_ alone did not affect the algal total lipid content under replete nutrient conditions, and this fact is in accordance to Gordillo et al. (2001b). However, we could prove that in the HL-population elevated pCO_2_ was interacting with temperature and that at low temperatures and elevated pCO_2_ the total lipid content decreased. The former showed that in *U. rigida* the composition of the phospholipids is changing following cultivation at elevated pCO_2_ and attributed this finding to a potential shift in the manner of carbon uptake. Since at low temperatures both the lipid composition of membranes changes (Floreto et al. 1993) and the contribution of the CCM to photosynthetic carbon supply decreases (Olischläger and Wiencke 2013b), our findings support this hypothesis. Again, the effect of elevated pCO_2_ on the lipid content of *S. latissima* is ecotype specific, with an insensitive Arctic population and a pCO_2_-sensitive temperate population.

### Ecological implications

Our results show that Arctic and temperate populations of *S. latissima* strongly differ in their BC and that the Arctic population is less susceptible toward ocean acidification (OA), but both ecotypes show a BC strongly affected by an increase in temperature.

Kelps act as host to other algae, animals, and microorganisms, and thus are providing a suitable habitat for a great variety of species (Bartsch et al. 2008). Beside the kelp itself, also the associated organisms are part of the linked heterotrophic food webs (Bartsch et al. 2008). Hence the expected change in C/N-ratio, carbohydrates, proteins, and lipids content due to global change would affect benthic food webs in the temperate and Arctic ecosystems. In the Arctic waters of Kongsfjorden (Spitsbergen), *S. latissima* is the preferred algae as food source for the abundant sea urchin *Strongylocentrotus droebachiensis* (Wessels et al. 2006), thus, this grazer and others would be affected by BC changes. In this regard, the increased C/N-ratio at high temperatures in both ecotypes might be important. Furthermore, since the C/N-ratio is only weakly impacted by OA but strongly by temperature, it is likely that global warming rather than OA might influence the benthic food web.

In conclusion, we demonstrate that ecotypes can significantly differ in their biochemical composition and in their susceptibility toward ocean acidification and temperature.

#### *Author contribution*

MO, CI, CW, and FJLG planned the experiments; MO and CI conducted the experiments and did the required measurements. MO did the statistical analysis of the data and wrote most parts of the manuscript with the assistance of all coauthors. All authors contributed to the writing process and all authors read and approved the manuscript.

## Abbreviations

BC: Biochemical composition
CCM: Carbon concentrating mechanism
pCO_2_: Partial pressure of CO_2_
HL: Helgoland
SP: Spitsbergen
